# Effect of obliquity of incident light on the performance of silicon solar cells

**DOI:** 10.1016/j.heliyon.2019.e01965

**Published:** 2019-07-02

**Authors:** R. Sharma

**Affiliations:** Department of Engg. Physics, Model Institute of Engg. & Technology (MIET), Jammu, India

**Keywords:** Energy, Materials science, Applied mathematics, Optics, Obliquity of sunlight, ARC, PC1Dmod, Solar cell, Reflectance, EQE, Conversion efficiency

## Abstract

The aim of this work is to investigate the effect of angle of incident light on the performance of silicon solar cell. In this regard, numerical calculations have been performed to obtain the reflectance for double layer antireflection coating (DLARC) of Si_3_N_4_ at various angles of incidence (i.e.$0^{o},15^{o},\;30^{o},\;45^{o},\;and\;60^{o}$) using transfer matrix method. Reflectances obtained, are found to increases with increase in angle of incidence. Calculated reflectances have been further used in the PC1D simulator as external reflectance files to study the performance of silicon solar cell. As a result of the simulation, the conversion efficiency (and short circuit current) of solar cell is found to decrease by 1.7% (0.062 mA/cm^2^) with increase in angle of incidence from $0^{o}\;to\;60^{o}$.

## 1 Introduction

Solar cell is a promising approach for terrestrial and space photovoltaic devices. But the main challenge regarding the performance of silicon solar cell is the reflection losses. When sunlight illuminate the front surface of solar cell, some part of light energy transmitted into the cell and get converted into electrical energy whereas some part reflects from the front surface. In order to reduce the loss due to reflectance on silicon surface, different methods have been used. Light trapping, surface texturing and anti-reflection coatings (ARC) are most widely used to reduce the loss due to reflection [1, 2, 3, 4, 5] (see Table 1).

**Table 1:** Value of short circuit current, open circuit voltage, fill factor and efficiency at various angles of incidence.

Angle (degree)	J_sc_ (mA/m^2^)	V_oc_ (Volt)	FF	η%
0	39.09	0.675	79.69	21.02
15	39.08	0.675	79.69	21.01
30	39.01	0.675	79.69	20.98
45	38.98	0.675	79.69	20.96
60	38.47	0.674	79.64	20.66

A set of well-designed antireflection coating (ARC) can reduce reflection from more than 30% (for bare silicon) down to less than 2% [5, 6]. Solar cells operate in wavelength ranging from 300 – 1200 nm. The ARCs containing single layer can be non-reflective only at single wavelength, generally at the mid of visible spectrum whereas ARCs containing double or more layers are effective over the whole visible spectrum [5, 6]. Many works have been reported on the antireflection coating with different materials such as SiNx/SiNx by R. Sharma [6], MgF_2_/SiNx by Dhungel et al. [7], SiO_2_/TiO_2_ by Lien et al. [8], Al_2_O_3_/TiO_2_ by Bahrami et al. [9], and MgF_2_/Ti_2_O_3_ by M. Medhat et al. [10].

Generally, efficiency of solar cell is calculated under normal incidence. But from sun rise to sun set, sun light is not always normal to the surface of solar cell. As a result degree of polarization of light changes with change in angle of incidence and this modify the reflective property of ARC. So, oblique incidence should also be considered while designing ARCs to improve the efficiency of solar cell. A very few reports on the oblique incidence of light are available [11, 12].

In this work an attempt has been made to investigate the effect of angle of incidence of sunlight on the performance of silicon solar cells with DLARC of Si_3_N_4_. The parameters of the silicon solar cell such as short circuit current (I_sc_), external quantum efficiency (EQE), and conversion efficiency are studied at various angles of incidence.

## 2 Theory

### 2.1 Theory of antireflection-coating (ARC)

**Fig. 1:**
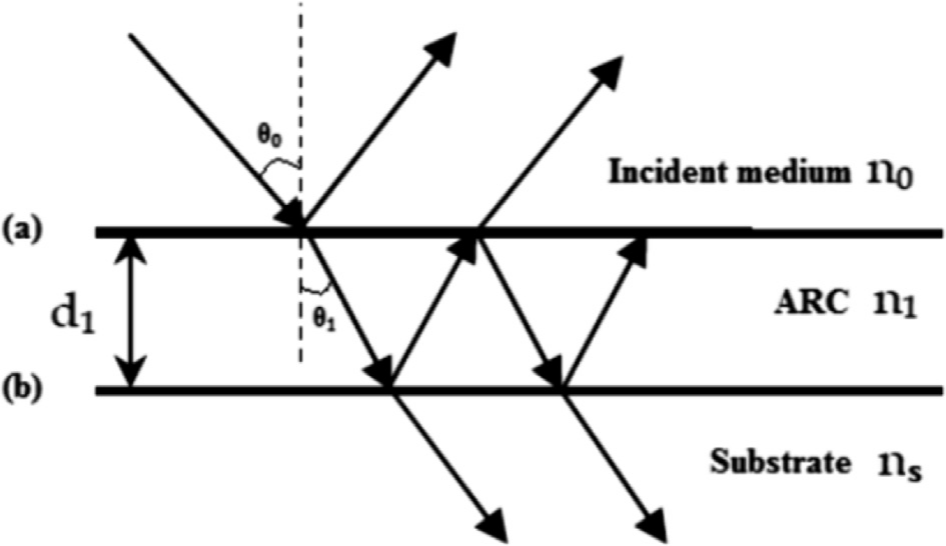
Reflection at interface between two media.

Most solar cells are coated with ARC layers to reduce reflection of light on the front surface of cell [5, 6, 7]. A good ARC is one that improves performance of solar cell by reducing reflection and increasing photocurrent [5, 6]. A set of well-designed ARCs on the front surface reduce the reflectivity on the front surface of cell from 30% down to less than 2% [4, 5]. Fig. 1 shows the principle of interference of light in thin film. The light reflected from boundary *a* and *b* interfere destructively and hence transfer energy to the solar cell [5].

Various methods are used to calculate the reflectivity of ARCs such as Fresnel formula, Rouard's method and transfer matrix method (TMM) [6, 10, 11]. But, transfer matrix method is the most commonly used as this relates the tangential components of electric and magnetic fields across the boundary of layers [11, 13]. For single layer system, field components at first boundary *a* are related to those at second boundary *b* by the expressions [13]:

(1) $$E_{a}=E_{b}cos\left(\delta \right)+H_{b}\left(\left(i\;\sin\;\delta \right)/\eta_{1}\right)$$

(2) $$H_{a}=E_{b}\left(i\eta_{1}\;\sin\;\delta \right)+H_{b}\;\cos\;\delta$$

These two equations in matrix form can be expressed as [13]:

(3) $$\left[\begin{array}{c} E_{a}\\ H_{a} \end{array}\right]=\left[\begin{array}{cc} \cos\;\delta & \frac{i\;\sin\;\delta }{\eta_{1}}\\ \eta_{1}\left(i\;\sin\;\delta \right) & \cos\;\delta \end{array}\right]\left[\begin{array}{c} E_{b}\\ H_{b} \end{array}\right]$$

where $\delta =\frac{2\pi n_{1}d_{1}\;\cos\theta_{1}}{\lambda }$ is the phase thickness of film, *d*_*1*_ is the thickness of film, *n*_*1*_ is the refractive index of film, *θ*_*1*_ is the diffraction angle related to the incidence angle *θ*_*o*_ by the Snell's law $\left(n_{o}\;\sin\theta_{o}=n_{1}\;\sin\theta_{1}\right)$ and *η*_*1*_ is the optical admittance. For *m* layer coating system, the overall transfer matrix is the product of individual transfer matrices, taken in order in which the light propagates through the multi-layer stake [13]:

(4) $$\left[\begin{array}{c} \;B\\ \;C \end{array}\right]=\left\{\overset{m}{\underset{k=1}{\prod }}\left[\begin{array}{cc} \cos\delta_{k} & \frac{i\;\sin\delta_{k}}{\eta_{k}}\\ \eta_{k}i\;\sin\delta_{k} & \cos\delta_{k} \end{array}\right]\right\}\left[\begin{array}{c} 1\\ \eta_{s} \end{array}\right]$$

where, $\delta_{k}=\frac{2\pi n_{k}d_{k}\;\cos\theta_{k}}{\lambda }$ (*k* = 1, 2, 3…….*m*) is the phase thickness of *k* – layers and *η*_*s*_ is the admittance of substrate. In case of oblique incidence, the admittance values of *s*-polarization and *p*-polarization are different. Thus for *k* – layers, they are [11, 12, 13]:

(5) $$\eta_{k}=\{\begin{array}{c} \;n_{k}\;cos\theta_{k}\;\;\;\;\;\;\;\;\;\;\;\;\;\;\;for\;s-polarization\\ \;n_{k}/cos\theta_{k}\;\;\;\;\;\;\;\;\;\;\;\;\;\;for\;p-polarization\; \end{array}$$

where $\theta_{k}$ can be obtained by the snell's law

(6) $$n_{o}\;\sin\theta_{o}=n_{k}\;\sin\theta_{k\;\;};\;k=1,\;2,\;3\ldots \ldots .m$$

The expression γ=C/B is the admittance for combination of multi-layer coating system [12, 13]. Thus, the reflectance for thin film system is

(7) R=|1−γηo1+γηo|2

For *R*_*s*_ component, *γ* and *η*_*o*_ in above equation should be replaced by *γ*_*s*_ and *η*_*os*_ while for *R*_*p*_ component, the corresponding *γ, η*_*o*_ should be replaced by *γ*_*p*_*, η*_*op*_. The total reflectance *R* is the average of *s* and *p* components:

(8) $$R=\frac{R_{s}+R_{p}}{2}$$

## 3 Design

**Fig. 2:**
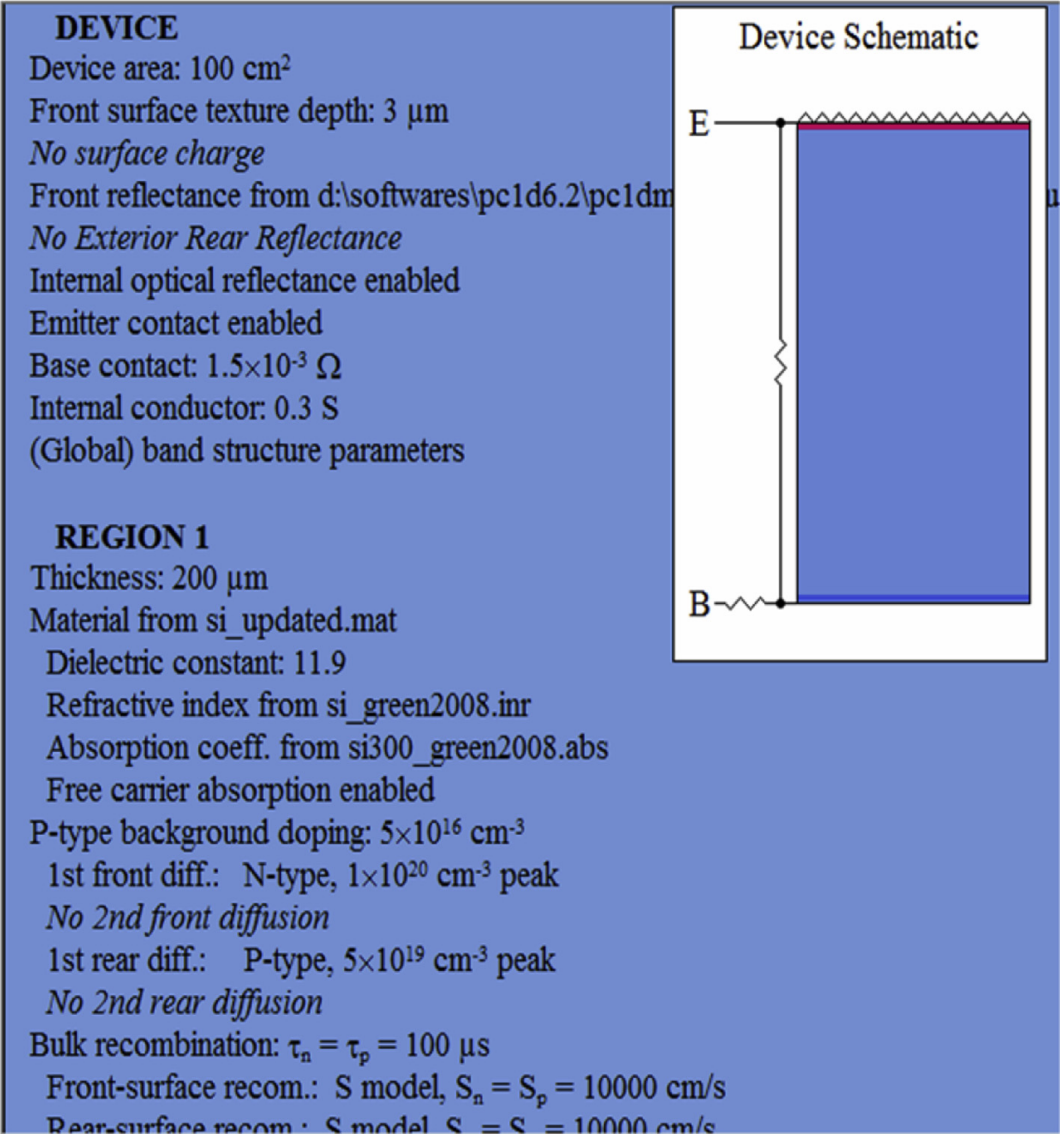
Summary of simulation parameters for PC1Dmod Model.

**Fig. 3:**
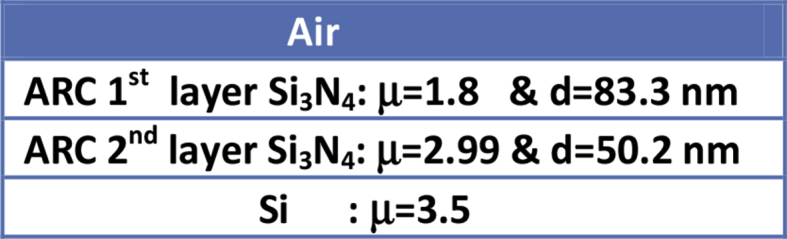
Structure of device used.

PC1D (mod 6.2) simulator is used to study the electrical and optical parameters of silicon solar cell. PC1D contains standard parameters which are used during simulation of solar cell. PC1D has two files “one-sun.exe” and “scan-qe.exe”. The file “one-sun.exe” gives short circuit current, maximum power and open circuit voltage while “scan-qe.exe” gives reflectance, internal quantum efficiency and external quantum efficiency verses wavelength. PC1D also has option to incorporate reflectance as an external file under “Front Reflectance”, which provides opportunity to include desired reflectance file. PC1D based simulation model of silicon solar cell is presented in Fig. 2. Si_3_N_4_ is used as antireflection coating with refractive index (and thickness) of top layer 1.8 (83.3 nm) and of bottom layer 2.99 (50.2 nm) [6]. Fig. 3 shows the structure device.

## 4 Results and discussion

### 4.1 Reflectance

**Fig. 4:**
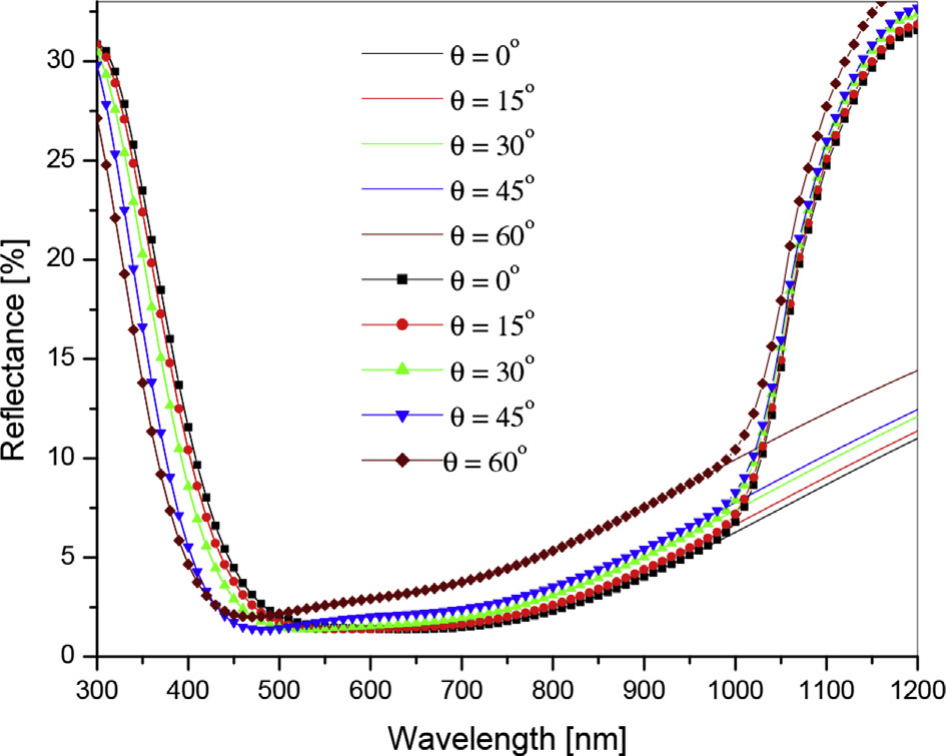
Reflectance spectra as a function of wavelength for various angles of incidence. Solid lined for numerically calculated (using TMM) and line plus symbol obtained from PC1D.

Fig. 4 shows variation of reflectance as a function of wavelength for silicon coated with double layer ARC of Si_3_N_4_. In this work anti-reflection coating has been designed to hold minimum reflection for the incident wavelength of 600 nm.

Reflectances for air/Si_3_N_4_/Si_3_N_4_/Si at incident angles $0^{o},15^{o},\;30^{o},\;45^{o},\;and\;60^{o}$ have been calculated numerically using TMM (equations 4–8). From plot (solid lines) one can observe that the reflectivity increases with increase in angle of incidence. For incident angle ranges from $0^{o}\;to\;45^{o}$, the variation in reflectance is less but at 60^o^ there is significant increase in the reflectance especially at larger wavelength range. Furthermore, the reflectances calculated above using TMM have been used in the PC1D simulator to study the effect of angle of incidence on the performance of the silicon solar cell. Fig. 4 show good agreement between the numerically calculated reflectance (solid lines) and that obtained from PC1D (line plus symbol curve).

### 4.2 External quantum efficiency (EQE)

The quantum efficiency of a solar cell is an important factor that relates optical parameter (reflectance) to electrical parameters (short circuit current and conversion efficiency) [14].

**Fig. 5:**
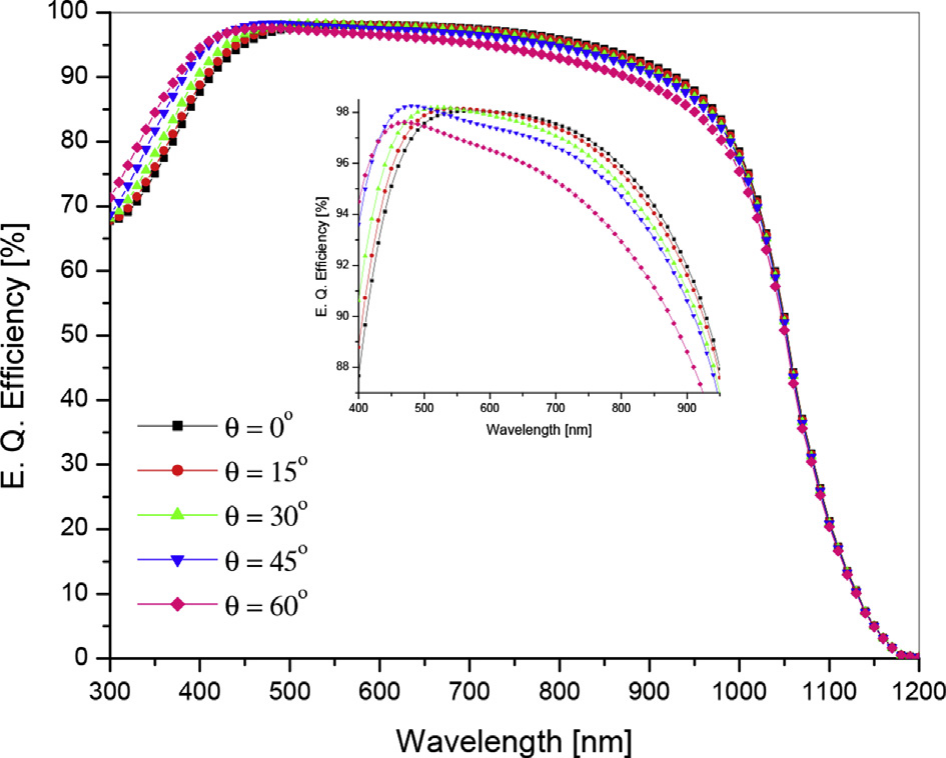
Variation of external quantum efficiency as a function of wavelength at different angles of incidence.

Quantum efficiency of a cell is the ratio of the number of carriers collected by the solar cell to the number of photons of a given energy incident on it. Thus the performance of a solar cell can be analyzed in terms of reflectance and EQE (i.e. if the reflectance is zero then EQE is 100%). EQE curves as a function of wavelength at angle of incident ranging from $0^{o}\;to\;60^{o}$ for a solar cell are shown in Fig. 5. Plot shows EQE decreases with increase in the angle of incidence. In small window of Fig. 5 one can observe EQE decreases significantly at larger angle (60^o^) especially at longer wavelength.

### 4.3 Electrical parameter

**Fig. 6:**
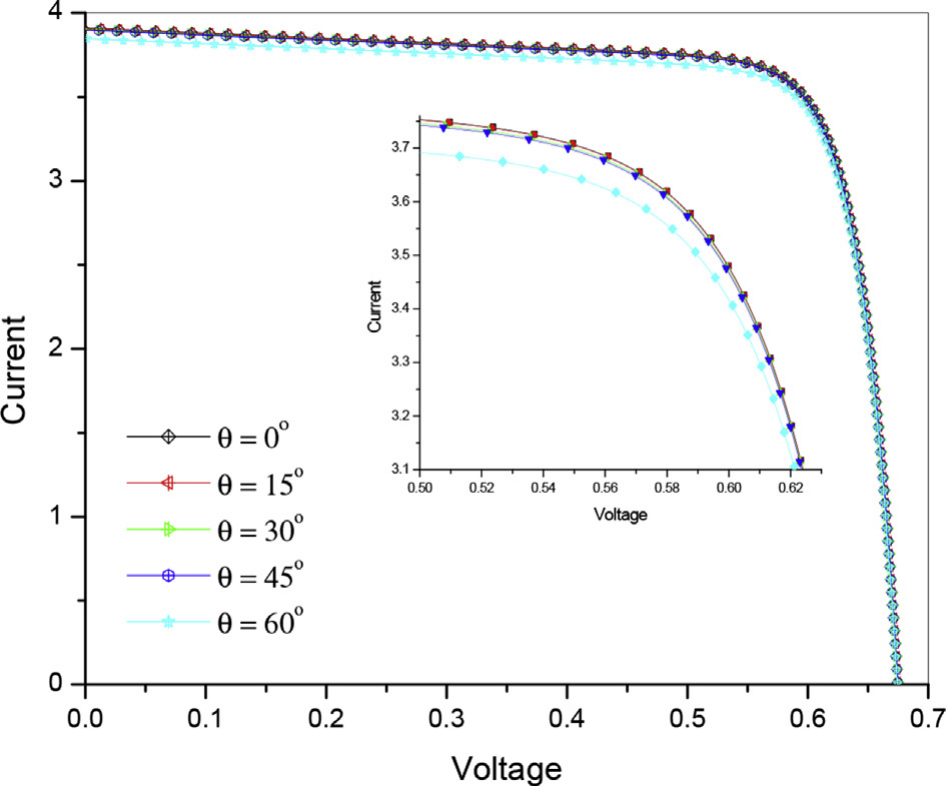
Current – voltage characteristics of silicon solar cell at various angles of incidence.

**Fig. 7:**
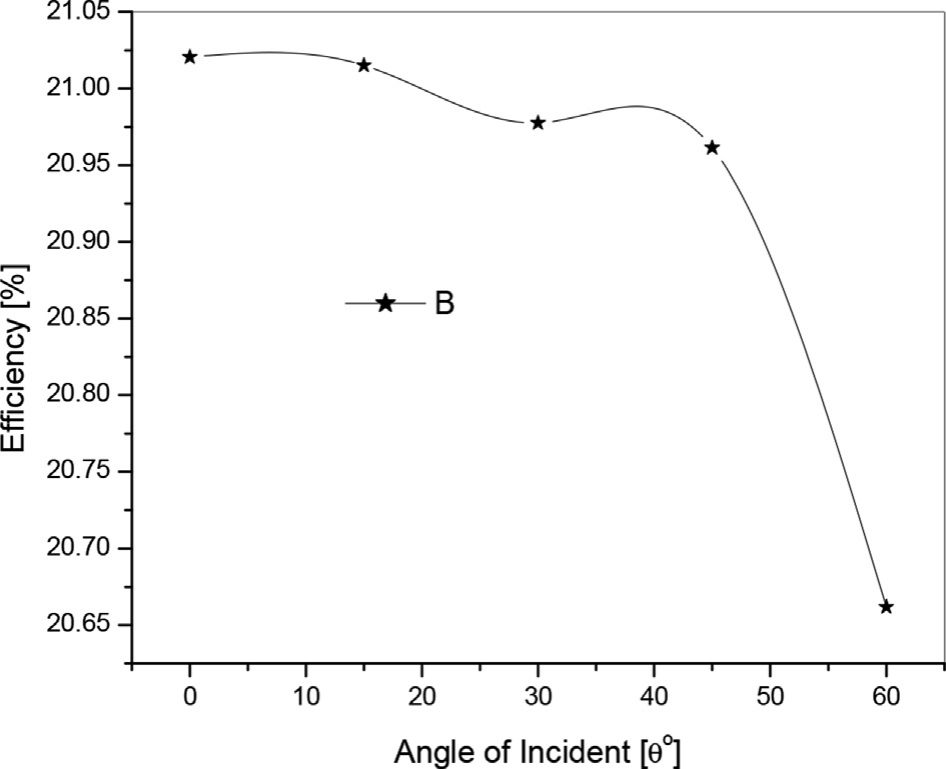
Variation of conversion efficiency of silicon solar cell as a function of angle of incidence of sunlight.

As a result of improvement in the optical parameters (i.e. reflectance & quantum efficiency), a good improvement is expected in the electrical parameters of the solar cell. As was expected from the analysis of reflectance and EQE that the short circuit current as well as conversion efficiency of solar cell will decrease with increase in angle of incidence of light. The I–V characteristic curves of the silicon solar cell at various angles of incidence are presented in Fig. 6, which shows that the change in the angle of incidence light changes the short circuit current due to effective photons absorption of incident light. Fig. 7 show the variation in efficiency of solar cell from 0^o^ to 45^o^ is very small (i.e. from 21.02% to 20.96%), but beyond 45^o^ there is an abrupt change in the efficiency of solar cell. Simulation shows the short circuit current decreases from 3.909 to 3.847 mA/cm^2^ whereas conversion efficiency of solar cell from 21.02 to 20.66% with increase in incidence angle from 0^o^ to 60^o^ respectively.

## 5 Conclusion

In present work, the effect of angle of incidence of sunlight on the performance of silicon solar was investigated. Results show that the conversion efficiency of silicon solar cell is almost constant for angle of incidence ranges from 0^o^ to 45^o^ and is decreased by 1.7% at angle of incidence 60^o^ with respect to 0^o^. So, it is concluded that the best performance of solar cell can be achieved between -45^o^ to +45^o^ angle of incidence of sunlight.

## Declarations

### Author contribution statement

Rajinder Sharma: Conceived and designed the experiments; Performed the experiments; Analyzed and interpreted the data; Contributed reagents, materials, analysis tools or data; Wrote the paper.

### Funding statement

This research did not receive any specific grant from funding agencies in the public, commercial, or not-for-profit sectors.

### Competing interest statement

The authors declare no conflict of interest.

### Additional information

No additional information is available for this paper.

## References

[1] Gangopadhyay U., Dhungel S.K., Basu P.K., Dutta S.K., Saha H., Yi J. (2007). Comparative study of different approaches of multicrystalline silicon texturing for solar cell fabrication. Sol. Energy Mater. Sol. Cell..

[2] Ju M., Gunasekaran M., Kim K., Han K., Moon I., Lee K., Han S., Kwon T., Kyung D., Yi J. (2008). A new vapor texturing method for multicristalline silicon solar cell application. Mater. Sci. Eng. B: Solid-State Mat. Adv. Tech..

[3] Basu P.K., Pujahari R.M., Kour H., Singh D., Varandani D., Mehta B.R. (2010). Impact of surface roughness on the parameters of industrial high efficiency NaOH-NaOCL textured multicrystalline silicon solar cell. Sol. Energy.

[4] Geng X.W., Li M.C., Zhao L.C. (2010). Research development of light trapping structure for thin film silicon solar cells. J. Funct. Mater..

[5] Sharma R., Gupta A., Virdi A. (2017). Effect of single and double layer antireflection coating to enhance photovoltaic efficiency of silicon solar. J. Nano- Electron. Phys..

[6] Sharma R. (2018). Silicon nitride as antireflection coating to enhance the conversion efficiency of silicon solar cell. Turk. J. Phys..

[7] Dhungel S.K., Yoo J., Kim K., Jung S., Ghosh S., Yi J. (2006). Double layer antireflection coating of MgF_2_/SiN_x_ for crystalline silicon solar cells. J. Korean Phys. Soc..

[8] Lien S., Wun D., Yeh W., Liu J. (2006). Tri-layer antireflection coating (SiO_2_/SiO_2_-TiO_2_/TiO_2_) for silicon solar cell using a sol-gel technique. Sol. Energy Mater. Sol. Cell..

[9] Bahrami A., Mohammadnejad S., Abkenar N.J., Soleimaninezhad S. (2013). Optimized single and double layer coating for GaAs solar cell. Int. J. Renew. Energy Resour..

[10] Medhat M., EL-Zaiat S., Farag S., Youssef G. (2016). Enhancing silicon solar cell efficiency with double layer antireflection coating. Turk. J. Phys..

[11] Beye M., Faye M.E., Ndiaye A., Maiga A.S. (2013). Optimization of SiN_x_ single and double layer ARC for silicon thin film solar cells on glass. Res. J. Appl. Sci. Eng. Technol..

[12] Chen F., Wang L. (2011). Chapter: Light Trapping Design in Silicon-based Solar Cells, in Tech.

[13] Macleod H.A. (2001). Thin Film Optical Filters.

[14] Sahouane N., Zerga A. (2014). Optimization of antireflection for industrial crystalline silicon solar cell. Energ. Procedia..

